# Post-mortem Cardiac MRI in Sudden Cardiac Death: The Interesting Intertwining of Radiology and Histology to Diagnose Arrhythmic Death or Myocardial Infarction

**DOI:** 10.2174/0115734056343601250123093759

**Published:** 2025-04-21

**Authors:** Giuseppe Bertozzi, Michela Ferrara, Aniello Maiese, Aldo Di Fazio, Donato Morena, Martina Padovano, Matteo Scopetti, Raffaele La Russa, Vittorio Fineschi

**Affiliations:** 1 SIC Medicina Legale Basilicata, Via Potito Petrone, 85100, Potenza, Italy; 2 Department of Anatomical, Histological, Forensic and Orthopedic Sciences, Sapienza University of Rome, 00185 Rome, Italy; 3 Department of Surgical Pathology, Medical, Molecular and Critical Area, Institute of Legal Medicine, University of Pisa, 56126 Pisa, Italy; 4 Department of Medical Surgical Sciences and Translational Medicine, Sapienza University of Rome, 00189 Rome, Italy; 5 Department of Clinical Medicine, Public Health, Life and Environment Science, University of L'Aquila, 67100 L'Aquila, Italy

**Keywords:** Post-mortem magnetic resonance, Cardiac diffusion tensor imaging, Fractional anisotropy, Sudden cardiac death, Arrhythmic death, Myocardial infarction

## Abstract

**Introduction:**

Although the “conventional” autopsy is still considered the “gold standard” for the definition of the cause of death, an emerging interest in non-invasive cadaveric investigations is spreading. Among all these, the application of post-mortem magnetic resonance imaging of the heart is increasingly gaining ground in the study of sudden cardiac death.

**Methods:**

Using the diffusion tensor imaging sequence, the present study aimed to demonstrate how through the fractional anisotropy value it is possible to qualitatively and quantitatively define sudden cardiac death, particularly in cases of sudden arrhythmic death syndrome. Four hearts were collected for the present pilot study: the first from a subject who died from a brain injury caused by a gunshot, and the other three hearts from subjects who died of sudden cardiac death. In all cases examined, the extracted hearts were hung inside a container containing 10% formalin solution and placed inside a 1.5T scanner with a 16-channel chest coil. Then, the cardiac diffusion tensor imaging sequence was performed and the quantitative maps of fractional anisotropy and apparent diffusion coefficient were obtained. After imaging analysis, the samples were processed, paraffin-embedded, and stained with hematoxylin and eosin and trichrome staining. Cases B, C, and D showed lower fractional anisotropy values than non-pathological one.

**Results:**

Histological investigation revealed extensive areas of fibrosis and foci of contraction band necrosis in heart B, myofiber disarray and interstitial fibrosis in heart C, and findings consistent with atonic death in heart D.

**Conclusion:**

The study aimed to demonstrate that in cases of sudden cardiac death, lower fractional anisotropy values, as already observed in clinical trials, are associated with arrhythmic heart disease or myocardial infarction. Quantitative, appreciable, and reproducible data could support such diagnoses.

## 1 INTRODUCTION

The autopsy, defined as “conventional”, is still considered the “gold standard” for the definition of the cause of death [1, 2]. The importance of autopsies has been described in numerous publications over the years. However, the rate of these investigations has declined globally, especially when clinical autopsy is considered. This decline is explained by specific cultural and religious reasons, lack of education in medical curricula, and financial aspects. The early phase of the recent SARS-CoV-2 pandemic, during which autopsies were initially prohibited in many countries due to fears of contagion, exemplified this [3, 4]. These factors have led to increased interest in alternative, non-invasive cadaveric investigations. This interest has been met with immediate response through imaging methods, like those used in clinical diagnostics for living patients. Over the years, these imaging techniques and protocols have been refined and implemented, becoming routine in some forensic pathology institutes worldwide for certain specific diagnoses [5-8]. Post-mortem computed tomography has proven to be very useful in traumatology, accurately identifying lesions that were later confirmed by autopsy. However, determining the vitality of these lesions is left to microscopic investigation [9-11]. Post-mortem magnetic resonance imaging, on the other hand, has allowed imaging to land in sudden-death territory. Sudden cardiac death, defined as “a rapid (without any specific chronologic limit) and unexpected or unforeseen — both subjectively and objectively — death which occurs without any clinical evaluation and in apparently healthy people in normal activity (primary or unexpected sudden death) or in patients in an apparently benign phase during the course of a disease (secondary or expected sudden death)” [12], represents a major public health problem. Thus, the Italian data provided by the National Institute of Statistics (called ISTAT from the Italian acronym), currently updated to 2021, identified cardiovascular diseases as the first cause of death (with 217,523 deaths in the period January-December 2021, compared to 174,511 from cancer and 63,915 from COVID-19 in the same period) [13]. The average annual incidence of sudden cardiac death across the European Union ranges between 36.8 per 100,000 and 39.7 per 100,000 individuals. The most frequent cause of sudden cardiac death in the adult population is coronary artery disease. For the younger population, the causes of sudden cardiac death are diverse and can be classified into structural (*e.g*., hereditary cardiomyopathies) and functional, resulting from arrhythmogenic conditions (including for illustration purposes: Brugada, long and short QT syndromes) [14, 15]. Thus, in the simplest cases, post-mortem examination reveals an underlying structural abnormality; however, a structurally normal heart is found in ≤59% of all sudden cardiac deaths in youth and ≤23% in athletes [16]. In these cases, in association with negative toxicology, a so-called sudden arrhythmic death syndrome is hypothesized. In this rugged terrain of intersection between sudden cardiac death and imaging, the previous studies of the present research group have demonstrated the possibility of studying *ex situ* hearts using post-mortem magnetic resonance [17, 18]. Subsequently, through post-mortem magnetic resonance imaging, it is possible to identify suspicious areas and then confirm by the histopathological investigation [19]. The present study aimed to compare, using cardiac diffusion tensor imaging sequences, the differences in fractional anisotropy and apparent diffusion coefficient values between a heart extracted from a subject who died from non-cardiac causes and the hearts of three subjects who died from sudden cardiac death. This could allow for a definition that is both qualitative and quantitative.

## 2 MATERIALS AND METHODS

### 2.1 Case Selection

Four hearts were collected for the present pilot study (A: 22-year-old male; B: 47-year-old male; C: 72-year-old male; D: 65-year-old male). Selection was made on an anamnestic basis, choosing a young subject who died of traumatic causes, an asymptomatic subject with a family history of sudden cardiac death, a patient with hypertension and coronary artery disease, and a patient with severe coronary artery disease. Heart A belonged to a subject for whom circumstantial and autopsy findings indicated death caused by a single gunshot wound resulting in brain injury. The other three hearts were from individuals who died of sudden cardiac death. In detail, Case B dealt with a subject who had no previous histories of hospital visits or pathologies known to his general practitioner or family members at the time of death that occurred at work; a positive family history of sudden deaths, however, was described. Case C, on the other hand, concerned an elderly man suffering from hypertensive heart disease who died at his home following an argument with his neighbor. Finally, Case D was a patient with severe coronary artery disease. Ethical review and approval were waived for this study, according to Italian legislation.

### 2.2 Imaging

In all cases examined, the autopsy was performed 24 hours after death and without the corpses having stayed in a cold room to minimize the bias of temperature variations (both due to alterations of the anatomical preparation and influences on the selected imaging technique). The extracted hearts were hung inside a container already prepared with a 10% formalin solution. This study was performed using a 1.5 T scanner (Philips Intera Achieva, Best, The Netherlands), 30 days after heart collection, with a 16-channel TORSO XL coil. An initial image was acquired to determine the short axis of the left ventricle. Then, the cardiac diffusion tensor imaging sequence was performed, and the quantitative maps of fractional anisotropy and apparent diffusion coefficient were obtained, using which 4 transmural regions (anterior, lateral, posterior, and septal) of the myocardium were studied.

### 2.3 Imaging Analysis

The elaborated protocol to study the signal behavior of fixed tissues, investigating mainly T1 and T2 weighted imaging, required 40 min. 3D isotropic imaging was preferred over bi-dimensional images because of post-processing reconstructions. Images were studied blindly (to the person and autoptic data) and after randomization by two operators with experience in cardiac radiology. A structured report was asked to be drafted [6]. The basis of the semiology of post-mortem magnetic resonance imaging analysis was assessed based on what was codified in a previous work [18]. The formalin fixation process, lasting 30 days, was chosen to ensure uniform tissue preservation and minimize the artifacts. Prolonged exposure to formalin is known to alter MRI parameters, particularly by increasing T1 relaxation times and reducing T2 signal intensity. Adjustments in sequence parameters were implemented to account for these variations. Additionally, specimens were imaged at a controlled room temperature (25-30 °C) to mitigate artifacts, though further studies are required to evaluate the effect of different fixation durations and temperatures.

### 2.4 Histological Examination

After the imaging analysis, the anatomical specimens were sampled following the “guidelines for the autopsy investigation of sudden cardiac death” [20]. Each sample was then processed and embedded in paraffin. Sections of 4-micron thickness were obtained from each sample and stained with hematoxylin and eosin; for cases B and C, additional sections were treated with trichrome staining. For each image, the magnification of the light microscopy reading was specified. The study was also completed by examination of the coronary tree. A 3 mm section was sampled alongside the entire course of the coronary arteries, including the surrounding epicardial tissue.

## 3 RESULTS

### 3.1 Cardiac Post-mortem Magnetic Resonance Imaging and Cardiac Diffusion Tensor Imaging

**Fig. (1):**
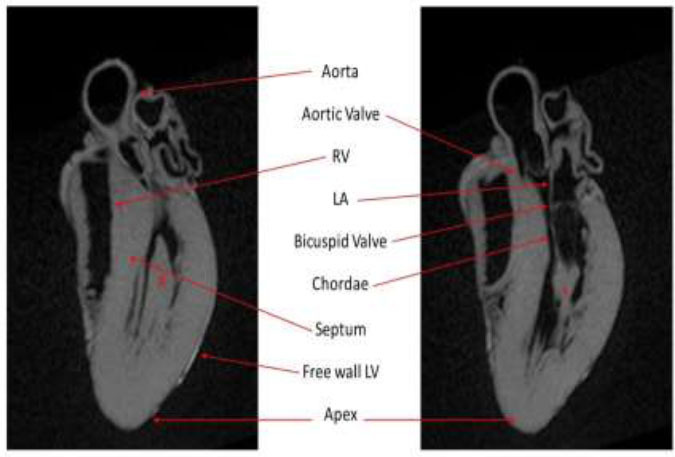
**Case A:** MPR T1-3D FFE (fast field echo); long axis - 2 chambers of healthy heart. *: anterior papillary muscle; #: posterior papillary muscle.

**Fig. (2):**
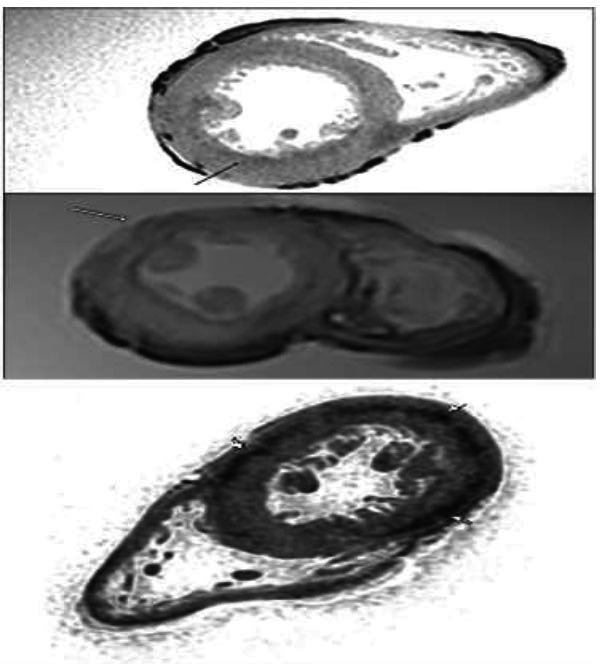
**T2-FFE. Case B** (above) presented a hemi-circumferential unevenly hypointense area at the mid-cardiac level, more evident along the left ventricle lateral and posterior wall, corresponding to extensive fibrotic areas at histological examination. **Case C** (middle) exhibited an in-homogeneously hypo-intense area on the epicardial side of the left ventricle anterior wall in the mid-basal area, as confirmed by fibrotic bundles at histological examination. **Case D** (below) demonstrated a hemi-circumferential unevenly hypointense area at the mid-cardiac level, along the left ventricle lateral and posterior wall.

The examination conducted on heart A revealed no alterations; indeed, it enabled us to perform morphological analysis of the heart (Fig. **1**). Cardiac post-mortem magnetic resonance imaging of Case B showed the following aspects: i) at long TR sequence, a fluid-sensitive hyperintense area with blurred edges in the mid-cardiac level and a sub-endocardial area of the left ventricle septum were observed; ii) at T2-FFE, a global reduction of the myocardial signal was documented; moreover, a hemi-circumferential unevenly hypointense area at the mid-cardiac level, diffuse but more evident along the left ventricle lateral and posterior wall, was noted (Fig. **2**). This last area was indicated as strongly suggestive of signs of scar due to a previous myocardial infarction, as highlighted by subsequent histological studies. Case C, on the other hand, exhibited the following i) hyperintense area in the STIR sequence both at the sub-endocardial level of the mid-cardiac area in the context of the left ventricle postero-lateral site and at the middle third of the septum, referring to clinical edema/hypercellularity, was observed; ii) at the T2-FFE sequence, an unevenly hypointense area was found at the level of the left ventricle lateral and posterior walls in the mid-basal cardiac region (Fig. **2**). Case D exhibited the following: i) at long TR sequence, a fluid-sensitive hyperintense area with blurred edges in the mid-cardiac level of the left ventricle was observed; ii) at T2-FFE, a global reduction of the myocardial signal was documented; moreover, a hemi-circumferential unevenly hypointense area at the mid-cardiac level, along the left ventricle lateral and posterior wall, was observed (Fig. **2**).

The results of the analysis of fractional anisotropy at the left ventricle wall are shown in Table **1**. The table outlines the fractional anisotropy and apparent diffusion coefficient values across myocardial regions, with standard deviations and variances included for clarity. The values highlight distinct patterns between pathological and non-pathological hearts.

### 3.2 Pathological Evaluation

In Case A, heart and coronary samples revealed no pathological results. Heart B showed the following macroscopic characteristics: 11.8x12x4.5 cm and 460 g, regular subepicardial fat. The semi-conservative technique dissection allowed to measure left ventricular anterior wall of 1.2 cm; left ventricular lateral wall of cm 1.45; left ventricular posterior wall of 1.4 cm; septum of 1.2 cm; right ventricular wall of 0.6 cm. Nothing was reported for the endocardium, which appeared translucent. Coronary arteries exhibited critical stenosis of all coronary tracts. The atrial chambers appeared to be of regular volume. All cardiac valves appeared thickened. Heart C was of normal shape and size: 12x11.5x7 cm and 550 g. An abundant representation of subepicardial fat was observed. The coronary study revealed diffuse critical stenosis. In this case, sampling with a semi-conservative technique made it possible to document a thickness of 1.5 cm at the left ventricular anterior wall; 1.9 cm at the lateral wall; 1.8 cm at the left ventricular posterior wall; 1.6 cm at the interventricular septum; 0.7 cm at the right ventricular area. Heart D measured 13x12x5.5 cm and weighed 560 g; the study of the coronary arteries detected critical stenosis of all tracts. The macroscopic sections followed the usual Virchow’s method modified by Prausnitz. The left ventricular chamber had an anterior wall of size 1.4 cm, a lateral wall of size 1.4 cm, a posterior wall of size 1.4 cm, and a thickness of interventricular septum equal to 1.4 cm. There was nothing reported for the right ventricular chamber, with walls being 0.7 cm thick and having regular volume. Numerous areas of bluish discoloration showed affected ventricular surface in its medial portion.

### 3.3 Histology

**Fig. (3):**
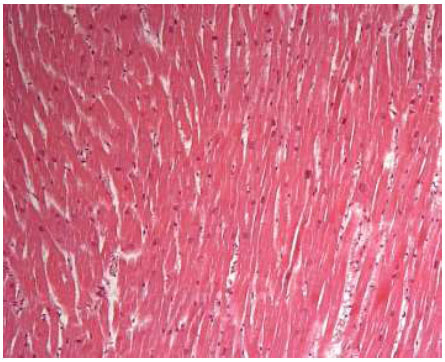
**Case A:** left ventricle wall characterized by notes of substantial normality (hematoxylin and eosin, 25x).

The microscopic study of Case A heart (Fig. **3**) highlighted the absence of alterations in the pericardial layers, which appeared of usual thickness, as well as the layer of subepicardial fat. The myocardium also showed myofibers of regular arrangement and coloration, with uniform and normal morphology nuclei.

**Fig. (4):**
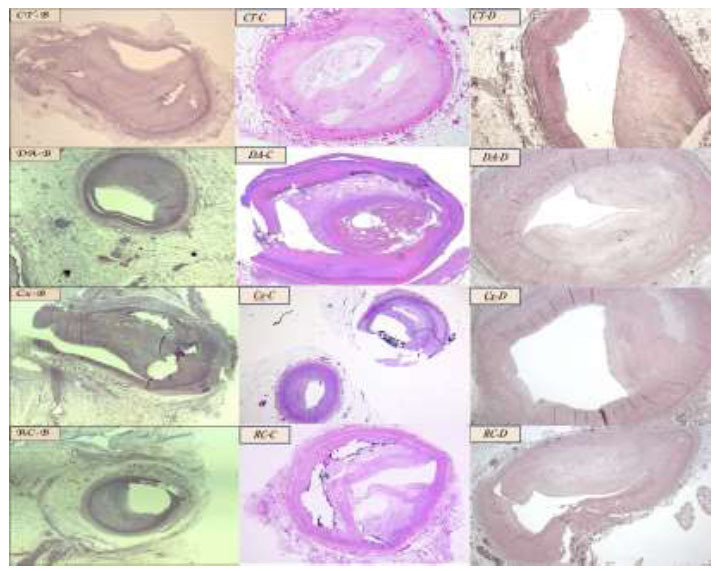
**Coronary sampling.** Common trunk (CT); left descending artery (DA); circumflex (Cx); right coronary (RC). All samples of cases B and C were photographed at 80x and stained with hematoxylin and eosin, except for Case C-RC, which was stained with trichrome (Azan stain). Microphotographs of Case D were observed at a magnification of 100x.

Microscopic observation of the coronary tree in cases B, C, and D showed severe luminal stenosis. Lumen reduction was calculated in each histologic section by assessing the average diameter of the residual lumen in percentage relative to the normal diameter measured on casts of that vessel preserved in maximal dilation in normal hearts (Fig. **4**).

**Fig. (5):**
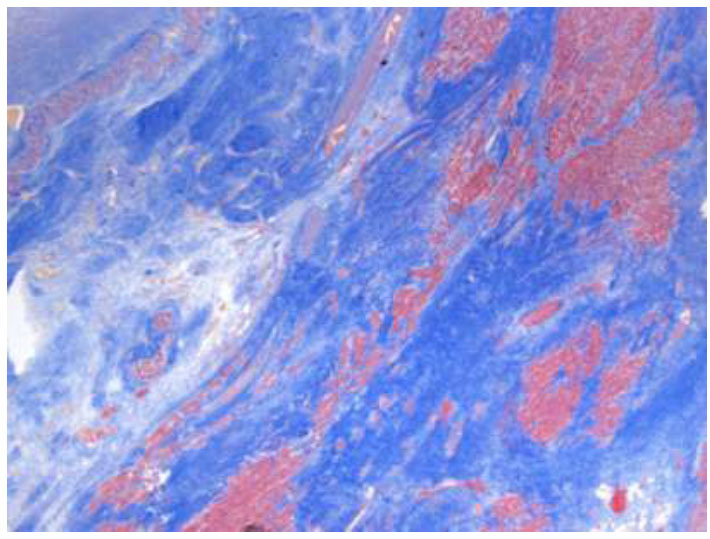
**Case B**: Left ventricular posterior wall with extensive fibrosis, evident in blue (Masson trichrome, 25x).

In Case B heart, multiple samples taken from the left ventricle showed extensive areas of interstitial fibrosis. In samples from the posterior, lateral, and septal walls of the left ventricle, entire areas of the myocardium were replaced by connective tissue, surrounding residual islets of myocellular bundles (Fig. **5**).

**Fig. (6):**
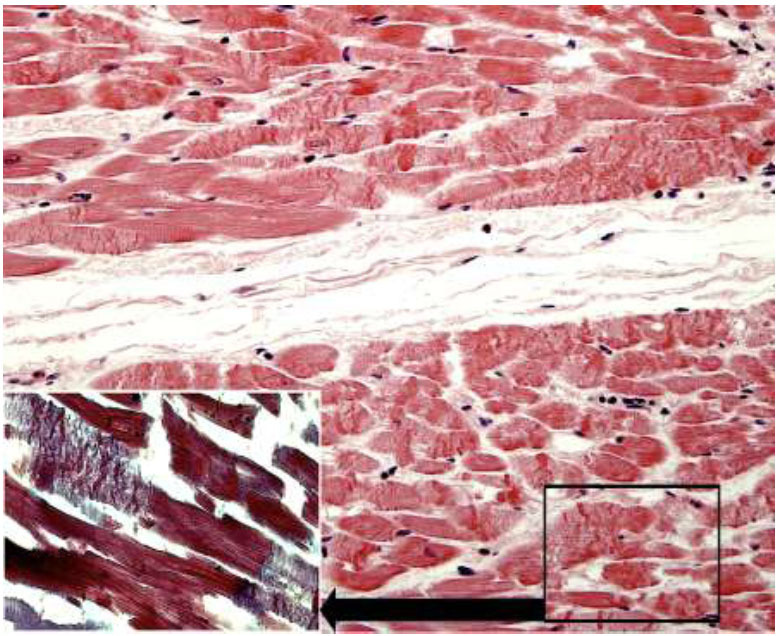
**Case B:** Left ventricular wall characterized by contraction band necrosis: pancellular lesion with fragmentation of hypercontracted myofibrils and band formation of hypercontracted or coagulated sarcomeres (hematoxylin and eosin 100x). (Insert in black) Pancellular and paradiscal lesion formed by about 15 hypercontracted sarcomeres without myofibrillar rhexis (hematoxylin and eosin, 100x). (Insert in white) The absence of edema, haemorrhage, and myofiber vacuolization was noted (Masson trichrome 250x).

In the most preserved areas, the residual muscular structure appeared to consist of normally arranged myocardial fibers of varying thicknesses, characterized by well-stained nuclei of varying sizes. These areas alternated with zones showing myofiber necrosis, along with hypercontracted myocytes with ruptured myofibrillar apparatus and the formation of transverse hypereosinophilic bands (Fig. **6**).

**Fig. (7):**
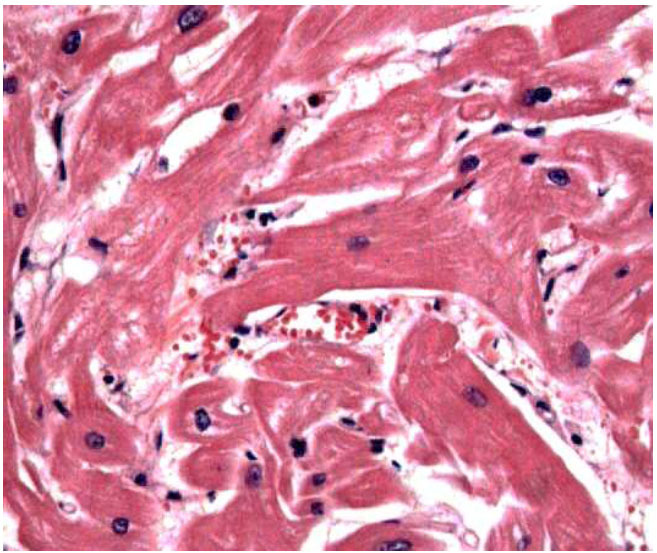
**Case C:** Left ventricular posterior wall characterized by myofiber disarray, the star-like disposition of adjacent myocytes, aligned obliquely or perpendicular to each other, and joined together by short, generally hypertrophic bridges, with interconnecting myofibrils and interstitial fibrosis (hematoxylin and eosin, 300x).

In Case C, the heart showed a widespread and generalized increase in the volume of the myofibers, which appeared to be characterized by clear hypertrophy of some nuclei. This finding was associated with aspects of disorganization and disorientation of the myofibers, oriented in multiple directions to assume, in some fields, a typical “ray-like” or “star-like” appearance (Fig. **7**).

**Fig. (8):**
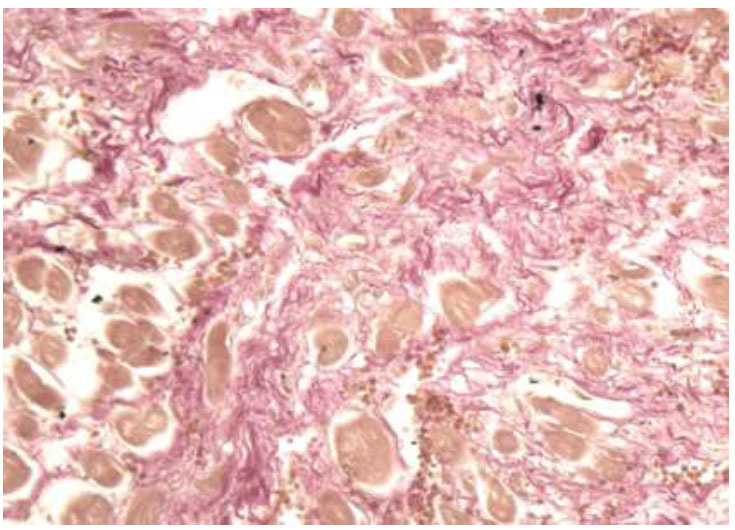
**Case C:** Left ventricular wall fibrosis evident in pink (Van Gieson trichrome, 100x).

Also noteworthy was the recurrence of areas of interstitial fibrosis, which take on a typically wavy appearance (Fig. **8**).

**Fig. (9):**
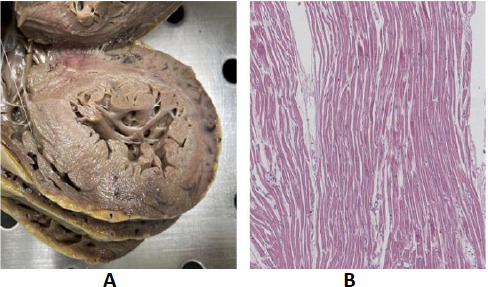
**Case D:** (**A**) Numerous areas of bluish discoloration showing affected ventricular surface in its medial portion. (**B**) Stretching of the myocardium in flaccid paralysis, resulting in a very early elongation of sarcomeres and nuclei (hematoxylin and eosin, 25x).

Case D exhibited infarct necrosis, characterized by an early disruption of the collagen matrix leading to the elongation of unprotected sarcomeres. This early stretching of myocardial cells and fibrillar disruption was attributed to intraventricular pressure (Fig. **9**).

In addition to the specific and more typical signs of these cases, foci of contraction band necrosis were also identified in the samples taken along the walls of the left ventricle of hearts C and D.

Thus, in the three “pathological” cases, the cause of death was attributed to acute cardiac arrest due to ventricular tachyarrhythmia in subjects affected by chronic ischemic heart disease (unknown in the first case and known in the second), and acute myocardial infarction in Case D.

## 4 DISCUSSION

Post-mortem magnetic resonance continues to demonstrate its usefulness in post-mortem studies. However, its limitations cannot be overlooked: the procedure is expensive, scan times are long, access to scanners is restricted, highly trained personnel with experience in post-mortem reporting are required, and no dedicated protocols are available. Furthermore, putrefaction and post-mortem cooling could alter the composition and structure of organs and consequently the quality of post-mortem imaging. However, this represents an important opportunity for developing new diagnostic protocols and semiotics in the post-mortem setting, which may require close collaboration between radiologists and forensic pathologists. Sudden cardiac death has always been challenging for forensic pathologists in reconstructing its phenomenology from the “*primum movens*” to the terminal event [21]. The certainty of the diagnosis is anchored to the histopathological investigation, especially in less evident cases where parenchymal alterations are not detectable by gross examination. However, its identification is not sufficient, in many cases, to clarify the chain of events leading to death. A classic example is found in cases of arrhythmic deaths, where contraction band necrosis is identified as a sign of adrenergic stress linked to malignant arrhythmias or ventricular fibrillation [21]. In contraction band necrosis, the myocardial cell stops in irreversible hypercontraction (tetanic death), as evidenced by the marked shortness of the sarcomeres, which have an extremely shorter length than that observed in physiological contraction and with characteristic abnormal and extreme thickening of the Z-lines. Such a lesion, in its early stages, cannot be detected by the naked eye or with routine staining methods. Later, myocardial cells coagulate and rupture, showing initial macrophage activity and the myocardium exhibits focal discoloration and depressions on its cut surface. In the healing phase, foci of fibrous tissue are observed [12]. The first histologic change, visible within 10 minutes of onset, is intense hypereosinophilia of hypercontracted myocardial cells with reticulation of the myofibrillar apparatus into abnormal and irregular pathological cross-fiber bands. These bands are formed by hypercontracted sarcomere segments with scalloped sarcolemma. Normal cells surrounding the hypercontracted ones take on a wavy appearance, and the spaces between the bands are filled with mitochondria. There is no evidence of platelet aggregation, other vascular changes, or interstitial or sarcolemma alterations. The characteristics of this form of myocyte necrosis are that it is multifocal, with multiple foci ranging from one or a few myocytes to thousands of myocardial cells, and it shows no site predilection as it is found in any cardiac region [12]. Two distinct morphological pictures are attributable to the described lesion. The first picture is a pan-cellular lesion consisting of fragmentation of the entire myocyte, which is matched by early rupture into pathological bands with total granular disruption (myofibrillar degeneration). The mechanism underlying these morphological changes is probably attributable to myocardial contraction acting on these rigid elements in tetany. The pancellular injury is repaired through macrophage digestion of all structures within the sarcolemmal tubes (alveolar pattern), followed by progressive collagenization [12]. The second morphological pattern, always associated with the previous one, is characterized by a single band of 10-20 hypercontracted sarcomeres near the intercalated disc (paradiscal lesion). This paradiscal lesion, always associated with the pancellular lesion, involves a unique band of hypercontraction affecting 10 to 15 sarcomeres adjacent to an intercalated disc without rupture. It features thin myofibrils and Z lines and often involves more than one myocyte. A hypercontracted center induces the waviness of normal adjacent myocytes, as observed by electron microscopy [12]. To illustrate the extent of this phenomenon, it should be considered that reperfusion strategies, the current standard therapy for acute myocardial infarction, can paradoxically lead to cardiomyocyte dysfunction known as reperfusion injury (resulting in arrhythmias, myocardial stunning, and microvascular obstruction), which can ultimately result in sudden death [22]. In these cases, it is necessary to resort to advanced immunohistochemical techniques to detect cell death, inflammation, neurohumoral activation, and oxidative stress [23]. In this work, these investigations were accompanied by genetics/genomics with the so-called molecular autopsy to identify alterations not only responsible for the cause of death, but also for individual susceptibility, compared to the general population [24, 25]. This plethora of investigations cannot fail to include forensic radiology. The rationale for the present pilot study stemmed from the premise that alterations induced by pathological states in otherwise normal heart tissue, such as fibrosis, contraction band necrosis, and fiber anisotropy, can be detected using a sensitive method, like MRI. Among magnetic resonance sequences, cardiac diffusion tensor imaging has been demonstrated to offer non-invasive microstructural evaluation, providing insights into cardiomyocyte organization [26]. Under physiological conditions, cardiac diffusion tensor imaging can detect the arrangement of cardiomyocytes in the left ventricle, which form a right-handed helix in the endocardium, a circumferential pattern in the mesocardium, and a left-handed helix in the epicardium, consistent with microscopic anatomy studies. Furthermore, cardiac diffusion tensor imaging was able to detect that the cardiomyocyte layers reoriented from a parallel direction in diastole to a perpendicular orientation in systole. This reorientation facilitates left ventricular wall thickening, which far exceeds that of individual cardiomyocytes [27-29]. Furthermore, preliminary *in vivo* studies have shown that cardiac diffusion tensor imaging can identify microstructural alterations in pathologies, such as amyloidosis [30] or hypertrophic cardiomyopathy [31]. In detail, diffusion imaging evaluates the diffusion of water, which is free, and when water molecules can spread equally in all directions (isotropic diffusion) [32]. Anisotropic diffusion, on the other hand, is typical of the myocardium since cardiomyocytes and connective tissues act as barriers, determining a restriction for impermeable barriers or an obstacle for barriers with different degrees of permeability. Furthermore, diffusion tensor imaging results are quantified by two main parameters: the apparent diffusion coefficient value, which refers to the overall tissue diffusivity, and the fractional anisotropy value, which reflects the directional dependence of the diffusion process and is represented by a scalar value. This value, representing the degree of anisotropy, has a value of 0 in the case of isotropic diffusion and a value of 1 in the case of diffusion limited to a single direction, assuming intermediate values as an expression of the anisotropic diffusion within the tissue being examined. Therefore, the more disorganized the tissue, the lower the fractional anisotropy value is expected. In these circumstances, even the diagnosis of arrhythmic death could be supported by a quantitative *datum*. Experimental studies on living animals have demonstrated the possibility of identifying different values between healthy hearts and pathological hearts [33]. However, to the best of our knowledge, this study is the first to be conducted not only in a post-mortem context but also on explanted hearts. The hypothesis has been confirmed in the present pilot study, revealing the fractional anisotropy values to be lower in pathological hearts than in healthy hearts. In common, however, it can be noted that fractional anisotropy values in healthy hearts *in vivo* and *ex vivo* are higher than pathological ones [33]. These data, also confirmed by histopathological examination, are in line with clinical studies [27, 34-38], in which it has been seen that the decrease in fractional anisotropy values occurs in infarct areas or thickening of the ventricular walls due to necrosis or cardiomyocyte disarray, expansion of the extracellular matrix, and replacement of normal tissue with less organized fibrosis [39-44]. The observed discrepancies between histological findings and MRI anomalies underscore the complexity of correlating microstructural changes with radiological signatures. For instance, in Case B, widespread fibrosis identified histologically did not fully correspond to the extent of anomalies detected in fractional anisotropy maps. This suggests that fractional anisotropy alone may not capture the complete spectrum of myocardial alterations, and complementary imaging techniques are required for a more nuanced interpretation. The cardiac diffusion tensor imaging sequence allows for the assessment of myocardial microstructure by quantifying water diffusion along preferential directions. *In vivo*, fractional anisotropy values are typically higher in healthy myocardium due to the organized alignment of myofibers, whereas pathological regions, such as those with fibrosis or myofiber disarray, exhibit reduced fractional anisotropy values. The results obtained, consistent with these *in vivo* observations, suggest that fractional anisotropy can serve as a robust marker for tissue integrity in post-mortem settings. However, differences in temperature, fixation methods, and post-mortem alterations must be accounted for when comparing these results. Although the present study focused on arrhythmic and ischemic causes of sudden cardiac death, the potential application of fractional anisotropy in identifying other etiologies, such as embolism or valvular pathologies, has not yet been explored. For example, integrating fractional anisotropy with advanced cardiac magnetic resonance imaging techniques, including late gadolinium enhancement or T1 mapping, may offer a more comprehensive assessment of myocardial abnormalities. Such advancements could facilitate earlier detection and differentiation of the mechanisms underlying sudden cardiac death.

## CONCLUSION

Conclusively, some authors have demonstrated how cadaveric cooling (the physiological algor mortis as well as the artificial cryopreservation) substantially influences post-mortem magnetic resonance imaging by prolonging the T1 value and shortening the T2 value, thus producing a reduction in the signal-to-noise ratio on both T1 and T2 sequences [45]. All these factors contribute to making post-mortem imaging different from ante-mortem clinical imaging and, consequently, require adjustments and modifications of the sequence acquisition parameters [17, 46-49]. This opens the scenario to how “conventional autopsy” should be gradually abandoned, and an increasingly integrated perspective of skills, a plurality of expertise, and multidisciplinary methods, always coordinated by the forensic pathologist, should be implemented [50-53]. This capability could enable increasingly precise responses to questions that, as recent history has shown, concern not only justice but also clinical practice, public health, and risk management, thereby restoring the role of autopsy in the medical field [54-59]. Thus, further studies are needed for the development of new diagnostic protocols and semiotics for post-mortem magnetic resonance [60-65]. Furthermore, in the present pilot study, case selection was based on primary pathological conditions rather than demographic uniformity. Age and medication history are recognized as potential factors influencing fractional anisotropy and apparent diffusion coefficient parameters due to their impact on myocardial tissue structure and diffusivity. To address these influences, partial mitigation was achieved by controlling environmental and procedural variables, including formalin fixation and imaging protocols. Future research involving a larger and more demographically uniform sample is planned to assess these potential confounding factors more comprehensively.

## Figures and Tables

**Table 1:** Left ventricle wall analysis. Fractional anisotropy value, representing the degree of anisotropy, has a value of 0 in the case of isotropic diffusion and a value of 1 in the case of diffusion limited to a single direction, assuming intermediate values as an expression of the anisotropic diffusion within the tissue being examined. Experimental studies on living human beings have demonstrated the possibility of identifying different values between healthy hearts and pathological hearts [1]. To confirm this also in a post-mortem setting, in the present study, the fractional anisotropy values were lower in pathological hearts compared to healthy hearts.

**Left Ventricle Wall**	Fractional anisotropy						Apparent Diffusion Coefficient			-		
**Case A**	**Case B**	**Case C**	**Case D**	**σ**	**σ^2^**	**Case A**	**Case B**	**Case C**	**Case D**	**σ**	**σ^2^**
**Anterior**	0,459±0,181	0,372±0,185	0,426±0,180	0,420±0,160	0,04	0,0019	0,741±0,500	0,900±0,502	0,714±0,442	0,840±0,528	0,10	0,0101
**Lateral**	0,452±0,178	0,364±0,177	0,436±0,195	0,426±0,180	0,05	0,0022	0,663±0,394	0,867±0,439	0,641±0,358	0,720±0,440	0,12	0,0155
**Posterior**	0,474±0,197	0,367±0,168	0,430±0,201	0,434±0,195	0,05	0,0029	0,620±0,360	0,853±0,426	0,712±0,448	0,670±0,360	0,12	0,0138
**Septal**	0,492±0,210	0,346±0,156	0,418±0,184	0,420±0,186	0,07	0,0053	0,620±0,385	0,804±0,357	0,850±0,531	0,720±0,444	0,12	0,0148

**Note: **σ (standard deviation); σ^2^ (variance).

## Data Availability

The data and supportive information are available within the article. Additional data can be obtained from the corresponding author [V.F] upon reasonable request.

## LIST OF ABBREVIATIONS

TR: Repetition Time
STIR: Short Tau Inversion Recovery
FFE: Fast Field Echo
RV: Right Ventricle
LA: Left Atrium
LV: Left Ventricle
MPR: MultiPlanar Reconstruction
CT: Common Trunk
DA: left Descending Artery
Cx: Circumflex artery
RC: Right Coronary artery
